# Pituitary-Directed Therapies for Cushing’s Disease

**DOI:** 10.3389/fendo.2018.00164

**Published:** 2018-05-01

**Authors:** Fabienne Langlois, Jennifer Chu, Maria Fleseriu

**Affiliations:** ^1^Department of Endocrinology, Centre Hospitalier Universitaire de Sherbrooke, Sherbrooke, Quebec, Canada; ^2^Department of Medicine, Division of Endocrinology, Diabetes and Clinical Nutrition, Oregon Health & Science University, Portland, OR, United States; ^3^Department of Neurological Surgery, Oregon Health & Science University, Portland, OR, United States; ^4^Northwest Pituitary Center, Oregon Health & Science University, Portland, OR, United States

**Keywords:** Cushing’s disease, hypercortisolemia, adrenocorticotropic hormone-secreting adenoma, pasireotide, cabergoline, roscovitine, retinoic acid, gefitinib

## Abstract

Cushing’s disease (CD) is caused by a pituitary corticotroph neuroendocrine tumor inducing uncontrolled hypercortisolism. Transsphenoidal surgery is the first-line treatment in most cases. Nonetheless, some patients will not achieve cure even in expert hands, others may not be surgical candidates and a significant percentage will experience recurrence. Many patients will thus require medical therapy to achieve disease control. Pharmacologic options to treat CD have increased in recent years, with an explosion in knowledge related to pathophysiology at the molecular level. In this review, we focus on medications targeting specifically pituitary adrenocorticotropic hormone-secreting tumors. The only medication in this group approved for the treatment of CD is pasireotide, a somatostatin receptor ligand. Cabergoline and temozolomide may also be used in select cases. Previously studied and abandoned medical options are briefly discussed, and emphasis is made on upcoming medications. Mechanism of action and available data on efficacy and safety of cell cycle inhibitor roscovitine, epidermal growth factor receptor inhibitor gefitinib, retinoic acid, and silibinin, a heat shock protein 90 inhibitor are also presented.

## Introduction

Clinical hypercortisolism is associated with significant morbidity and mortality (1). The vast majority of cases of endogenous Cushing’s syndrome are caused by a pituitary corticotroph neuroendocrine tumor; Cushing’s disease (CD). Surgery is the mainstay of therapy in most cases (2). However, 20–40% of patients, depending on tumor size and surgeon expertise, will not be cured by transsphenoidal surgery, and for patients in remission, 20–35% will relapse within 10 years (3–5). Options include medical treatment, which has a significantly increased role over the last several years, repeat surgery, radiation, and bilateral adrenalectomy. From an initial disease description by Cushing (6), advances in understanding corticotroph adenoma pathology have hugely increased, more so in the last decade (7–9). Today, accumulated CD knowledge allows for development of precise molecular targeting for adrenocorticotropic hormone (ACTH) secretion and cell proliferation. Future advances may also contribute to development of medication for other pituitary neuroendocrine tumors for which few options exist, for example, silent corticotroph tumors (10). In this review, we will discuss the array of pituitary-directed medical therapies for CD, including approved medications and new medical therapies on the horizon.

## Historical Review of Pituitary Targeted Drugs

Largely abandoned over the years, it is important to review targets and/or pathways for some previous pituitary targeted drugs. Peroxisome proliferator-activated receptor gamma(γ) agonists, mostly utilized and abandoned as an antidiabetic therapy, have been attempted in treatment of Nelson syndrome and CD with controversial results (11–13). Peroxisome proliferator-activated receptor-γ nuclear receptors are highly expressed in ACTH adenomas; however, high doses were necessary to induce an antiproliferative effect and ACTH inhibition; and escape phenomenon was frequent (14, 15). Valproic acid inhibits gamma-aminobutyric acid (GABA) aminotransferase, increasing GABA, thus leading to an inhibitory effect on ACTH release, yet, placebo controlled studies failed to demonstrate efficacy (16). Cyproheptadine is an anti-serotonergic, histaminic and cholinergic agent. Since ACTH may be under serotoninergic control, cases reports of response were described (17, 18), but data consisted mostly of unsuccessful trials (15). Other serotonin antagonists have been also previously studied, however, with limited results (19). Finally, in murine and human pituitary tumors, doxazosin decreased tumor growth and ACTH levels (20), but no evidence actually support clinical use of alpha-1 adrenergic receptor antagonists in CD.

## Medications Approved for CD

### Somatostatin Receptor Ligands (SRLs)—Pasireotide

**Figure 1:**
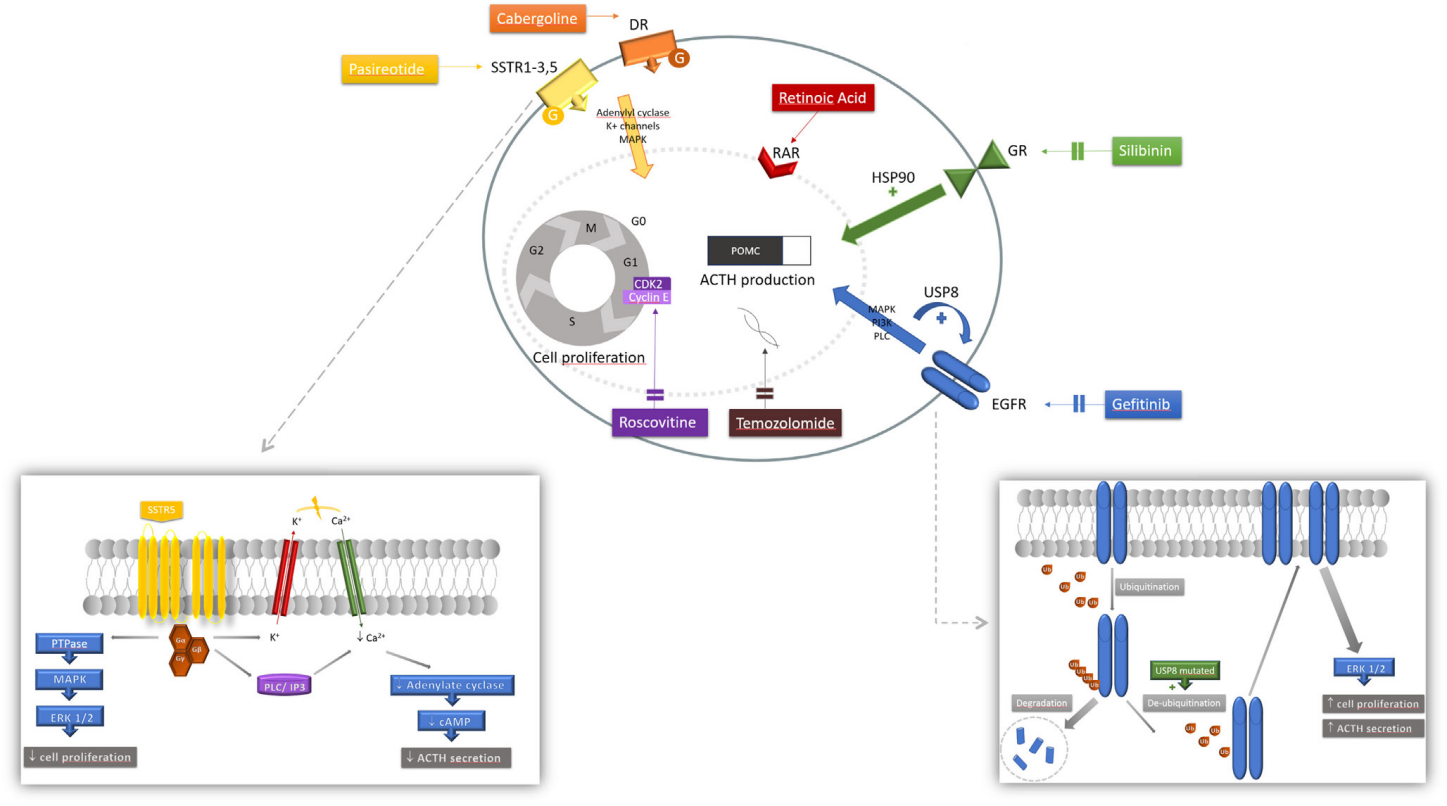
Mechanism of action of pharmacologic therapies. Various mechanisms of action of medications acting at the corticotroph tumor are represented. Each agent involves a different cascade of action resulting in both inhibition of pro-opiomelanocortin (POMC) and adrenocorticotropic hormone (ACTH) synthesis and secretion, and tumoral cell proliferation. Pasireotide is a ligand for somatostatin receptor (SSTR) 1, 2, 3, and 5, activating a G-protein-coupled receptor. Signaling pathways include PTPase and downstream mitogen-activated protein kinase (MAPK) and extracellular signal-regulated kinase 1/2 (ERK 1/2) inhibiting tumorigenesis; and also closure of the potassium (K^+^) voltage channel and activation of the phospholipase C (PLC) and inositol triphosphate (IP3) pathway inducing calcium (Ca^2+^) influx that will decrease cyclic AMP (cAMP) formation and ACTH secretion. Cabergoline is a dopamine receptor (DR) agonist, binding to a G-protein-coupled receptor activating adenylate cyclase, MAPK and K^+^ efflux. Retinoic acid binds to its nuclear receptor [retinoic acid receptor (RAR)] to induce its inhibitory effect. Silibinin induces conformation changes at the glucocorticoid receptor (GR) level by inhibiting heat shock protein 90 (HSP90); net effect is an increase sensitivity to circulating corticosteroids restoring glucocorticoid negative feedback inhibition. Upon ligand binding, epidermal growth factor receptor (EGFR) induces tyrosine kinase activity with downstream MAPK, phosphatidylinositol-3-kinase (PI3K) and PLC signaling pathways. Ubiquitination of the internalized receptor targets the EGFR to be degraded in the lysosome. In ubiquitin-specific protease 8 (USP8)-mutated tumors, the USP8 mutation increases de-ubiquitination thus decreasing EGFR degradation. More EGFR are found at the membrane increasing its stimulatory effect, partly mediated by ERK 1/2 pathway. Gefitinib effectively blocks EGFR to decrease its activity.

Somatostatin (SST) is an inhibitory polypeptide hormone with ubiquitous receptors (SSTR) and pleiotropic actions (21). Corticotroph adenomas express mainly SSTR subtypes 5 and 2 (21, 22). Pituitary SSTR5 expression seems to be unaffected by high cortisol levels, whereas expression of SSTR2 is suppressed, but can upregulate with eucortisolemia (21, 23). The activated SSTR decrease cyclic adenosine monophosphate and increase potassium efflux, preventing ACTH release. In addition, SSTR are G-protein-coupled receptors and downstream effects of the SSTR2 and SSTR5 receptors encompass Ras–Raf mitogen-activated protein kinase (MAPK) and extracellular signal-regulated kinase 1/2 pathways, leading to cell growth arrest (23–25) (Figure 1).

Pasireotide is a SRL with 40 times the binding affinity to SSTR5 compared with octreotide; it also has high affinity for SSTR1, 2, and 3 (21, 23). Treatment with pasireotide has been shown to restore SSTR2 membrane density thus improving drug efficacy (26). Pasireotide is now approved for subcutaneous administration twice a day (27, 28) in many countries around the world; bioavailability is excellent and half-life with subcutaneous administration is approximately 12 h (29). Long-acting pasireotide LAR in CD (10 and 30 mg once a month administration) has also been studied in a large clinical trial (30).

## Biochemical Control and Clinical Improvements

**Figure 2:**
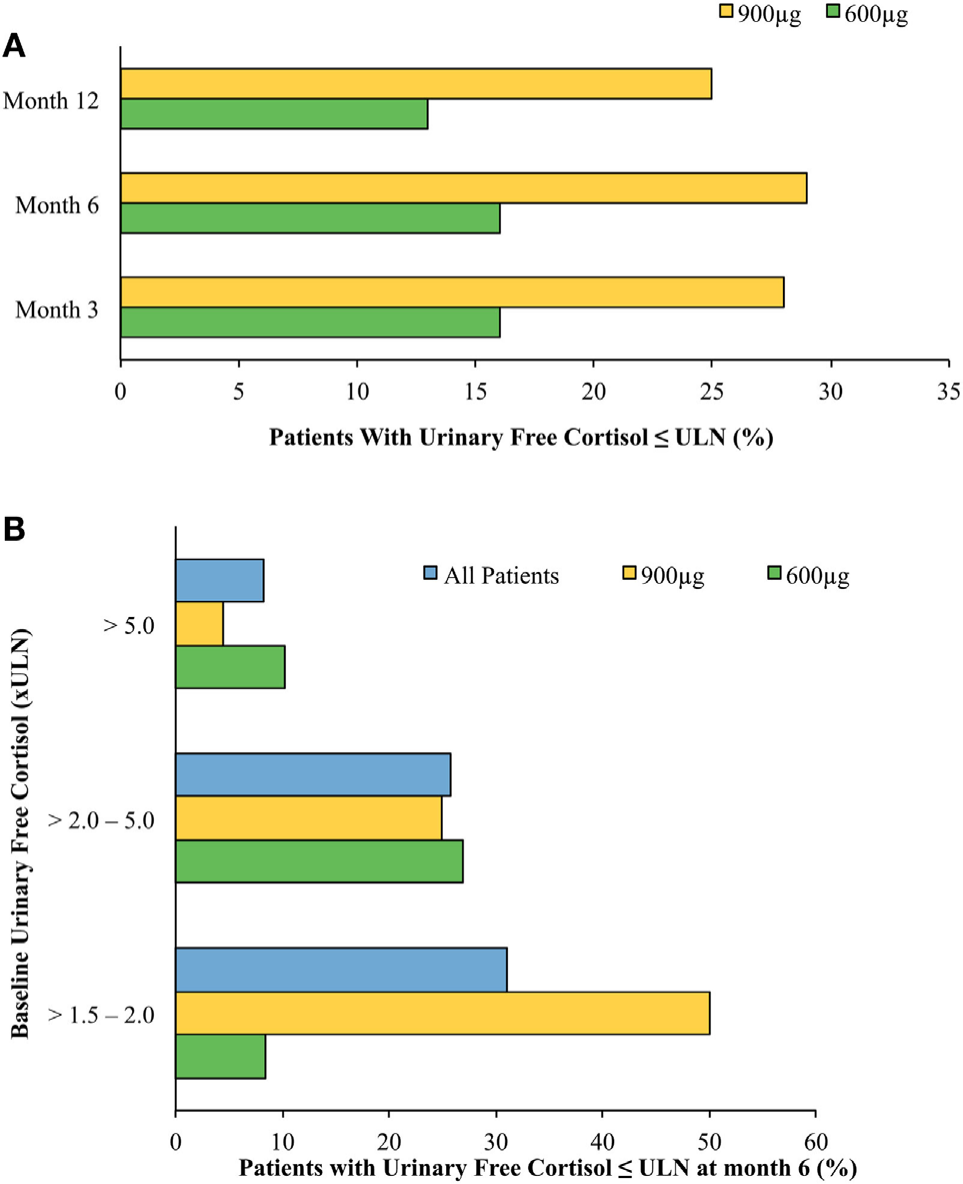
Normalized mean urine free cortisol (mUFC) in patients treated with short-acting pasireotide (600 or 900 μg twice daily) (28). **(A)** Cushing’s disease (CD) remission by treatment arm by month. Patients treated with twice-daily subcutaneous pasireotide obtained normalization of mUFC at months 3, 6, and 12. Patients’ dosages were up-titrated beginning at month 3. Patients who were on higher pasireotide doses regardless of treatment arm showed better response. Patients who had greater than five times the upper limit of normal (ULN) mUFC were more likely randomized to the 600 μg twice-daily treatment arm, possibly accounting for discrepancies between treatment arms’ responsiveness to pasireotide. Patients with uncontrolled hypercortisolism were more likely to stop the study. **(B)** CD remission at month 6 stratified by baseline urinary free cortisol (UFC). Stratification of patients by baseline mUFC predicted response to treatment with twice-daily subcutaneous pasireotide. The lower the patient’s baseline mUFC, the more likely they were to obtain normalized free UFC levels. As above, patients who had greater than five times the ULN mUFC were more likely randomized to the 600 μg twice-daily treatment arm.

A phase II trial suggested efficacy of pasireotide in CD (27), and a large phase III clinical trial by the Pasireotide B2305 Study Group followed (28). The phase III trial lasted 12 months and enrolled 162 patients, randomized to 600 or 900 μg subcutaneously, twice daily. A biochemical response [decrease in 24 h urinary free cortisol (UFC)] was robust and observed within the first month on therapy. At the end of the study, respectively, 13 and 25% of patients treated with 600 or 900 μg twice daily normalized their UFC (Figure 2). Of note, baselines UFCs were significantly higher in the 600 μg group (730 compared with 487 nmol/24 h in the 900 μg group), which may have attenuated results in the latter. An additional 16% in the 600 μg group decreased their UFCs by more than 50% at 12 months. In brief, at 6 and 12 months, approximately 30–40% of patients had partially or completely controlled disease (Figure 2). In the study, patients who did not respond within 2 months had a high likelihood of being non-responders. In addition, a less severe disease, e.g., lower baseline UFC, also predicted response. Morning ACTH, late-night salivary cortisol (LNSC) and serum cortisol concentrations also changed in parallel with a decrease in UFC. Significant improvement in multiple clinical parameters, including systolic and diastolic blood pressure, weight, waist circumference, lipid profile, depression, and quality of life were noted [Table 1a,b (28, 30)]. Interestingly, significant clinical improvement has been noted even in patients without UFC normalization (31). However, the study had a high discontinuation rate of more than 50% at 12 months, withdrawal being mostly in patients with an unsatisfactory therapeutic effect.

**Table 1:** Clinical improvement in Cushing’s disease (CD) patients treated with pasireotide.

(a) Overall changes in clinical signs and symptoms of CD when treated by pasireotide (16, 30, 31)					

	Pasireotide subcutaneous, twice daily			Pasireotide LAR	
	Overall average		Median	Overall average	
	6 months (*n* = 107)^a^	12 months (*n* = 78)^a^	60 months (*n* = 16)^a^	7 months (*n* = 116)^a^	12 months (*n* = 104)^a^
Weight, kg	−4.4	−6.7	−6.2	−3.3^b^	−5.0^b^
Body mass index, kg/m^2^	−1.6	−2.5	−2.3	−1.0^b^	−2.0^b^
Waist circumference, cm (*n*)	−2.6	−5.0 (69)	–	−4.5	−5.4
Systolic blood pressure, mmHg	−9.1	−6.1	−4.3	−5.6^b^	−4.8^b^
Diastolic blood pressure, mmHg	−4.6	−3.7	−1.7	−3.8^b^	−3.2^b^
Total cholesterol, mg/dL	−14.5^b^	−20.7^b^	−50.3^b^	−17.3^b^	−13.5^b^
Low-density lipoprotein, mg/dL	−11.6	−15.5	−27.1	−15.5^b^	−13.6^b^
High-density lipoprotein, mg/dL	–	–	−3.6	−1.8^b^	−0.4^b^
Triglycerides, mg/dL	0	−17.7	−26.6	−9.5^b^	−4.6^b^
Health-related quality of life score, in points	+9.5	+11.1	–	+6.8^b^	+6.7^b^
Tumor volume, % change from baseline, cm^3^ (*n*)	−3.64% (75)	−27.14% (75)	+0.006% (6)	–	−17%
Facial rubor, % patients with improvement (*n*)	46% (96)	50% (69)	80%	44% (108)	44% (86)
Supraclavicular fat pad, % patients with improvement (*n*)	41% (93)	54% (68)	85%	34% (108)	38% (86)
Dorsal fat pad, % patients with improvement (*n*)	39% (93)	55% (67)	60%	35% (107)	39% (85)

**(b) Improvements in selected parameters at month 6 when treated with pasireotide subcutaneously twice daily compared with baseline^c^ (16)**					

	**Baseline (*n* = 162)**	**Controlled (*n* = 32)**	**Partially controlled (*n* = 22)**	**Uncontrolled (*n* = 62)**	

Weight, kg	81.6	−5.6	−3.2	−4.1	
Body mass index. kg/m^2^	30.3	−2.1	−1.2	−1.5	
Systolic blood pressure, mmHg	133.5	−13.4	−7.5	−7.3	
Diastolic blood pressure, mmHg	86.3	−7.7	−3.9	−3.2	
Total cholesterol, mg/dL (*n*)	224.0	−23.2 (31)	−11.6	−11.6 (61)	
Low-density lipoprotein, mg/dL (*n*)	135.3	−15.4 (31)	−3.9	−11.6 (61)	
Triglycerides, mg/dL (*n*)	159.4	−8.9 (31)	−17.7	−17.7 (61)	
Health-related quality of life score, in points (*n*)	41.1	+9.6 (31)	+8.9	+9.7 (61)	

*^a^Unless otherwise stated*.

*^b^Values are based on calculated weighted averages of data provided in the primary articles*.

*^c^Controlled patients achieved urinary free cortisol (UFC) ≤ upper limit of normal (ULN), partially controlled patients had UFC ≥ ULN but with >50% decrease from baseline, and uncontrolled patients did not have UFC ≤ ULN and who did not achieve >50% decrease from baseline. Regardless of their status, all groups demonstrated tangible improvements in blood pressure, weight, BMI, lipid profiles, and quality of life score*.

Similar results were obtained in the recently published pasireotide LAR study (30). UFC normalization was reached in 50% of patients with mild disease [UFC 1.5–2 times upper limit of normal (ULN)] and one-third of patients with UFCs 2–5 times normal. Based on previous results of possible lower efficacy, patients with UFC > 5 ULN were excluded from this study. Of note, those two studies used different UFC assays but each study used one assay consistently within its respective methodology. The 5-year follow-up study showed that short-acting pasireotide responders had continued improvement in clinical parameters at month 60 (32). The response to pasireotide treatment is thus usually long lasting (32, 33). However, loss of response was observed in few patients with ACTH-producing invasive macroadenomas, in some occurring after treatment interruption (34, 35). This may reflect fluctuations in underlying tumor secretion, or an escape phenomenon of unknown patho-physiology.

## Tumor Size

Of the patients who had measurable tumors as assessed by pituitary magnetic resonance imaging, treatment with subcutaneous pasireotide had demonstrable decrease in tumor volume at month 12 (mean decrease of 9% for 600 μg group compared with 44% in the 900 μg group) (28). In the pasireotide LAR trial, decreased tumor volume was also observed in the two groups (17.8 and 16.3% in the 10 and 30 mg group) (30). Effects on tumor size are likely due to interference with cell growth and cell cycle (25, 36). Another possible mechanism may be inhibition of vascular endothelial growth factor secretion, as demonstrated in non-functioning pituitary adenomas treated with pasireotide (37).

## Adverse Events

The most prominent adverse effect with pasireotide treatment is hyperglycemia. Both fasting and postprandial glucose concentrations were elevated in a dose-dependent manner in both phase II and III trials (27, 28). The mechanism in healthy volunteers seems to be a decrease in insulin and incretins (gastric inhibitory polypeptide and glucagon-like peptide-1) production rather than a change in insulin sensitivity *per se* (38). HOMA-β calculations and fasting insulin levels in a small study demonstrated insulin secretion decreased by nearly 50% after 12 months’ pasireotide usage in patients with CD (*p* = 0.015; *p* = 0.007, respectively). However, euglycemic hyperinsulinemic clamp evaluation did not demonstrate differences in insulin sensitivity before or after initiation of pasireotide (39). Glucagon levels were also decreased on therapy, but to a lesser extent (40). More than 70% of patients in both pasireotide twice daily and LAR studies (41), and nearly all patients followed for 5 years, had a hyperglycemia-related adverse event (32). Patients with preexisting diabetes generally needed at least one additional antidiabetic medication and glycemic control was readily obtained. Approximately 75% of patients with prediabetes progressed to diabetes. Nearly one-third of patients who had normal glucose tolerance developed prediabetes, and another 50–60% developed frank diabetes at one point in the study (27, 28). Fasting plasma glucose levels rose between 20 and 35 mg/dL after initiation of pasireotide and remained elevated at month 12 (31). HbA1c rose by 1–2% points by month 6 on pasireotide twice daily and persisted through month 60 (31, 32). This probably reflects the integration of glucose homeostasis changes by an improvement of glucose sensitivity with CD treatment and pasireotide-induced hyperglycemia.

Close observation and rapid management of hyperglycemia in patients with CD who have insulin resistance at baseline and develop hyperglycemia or diabetes after therapy initiation with pasireotide is essential. Furthermore, diabetic patients should have their control optimized before initiation of pasireotide. Non-diabetic patients should undergo self-monitoring of blood glucose at least twice a week in first week, then once weekly thereafter (41). Glucose-lowering agents of choice are metformin (effect probably *via* increase in GLP-1) and a dipeptidyl peptidase 4 inhibitor or GLP-1 agonist, due to the described mechanism above (41). Notably, hyperglycemia is reversible after stopping therapy (41) and patients need to be followed closely after stopping pasireotide and antidiabetic medications need to be adjusted to avoid hypoglycemia. Studies to detect optimal therapy for hyperglycemia associated with pasireotide in patients with CD are ongoing (42).^1^

Other common side effects, similar to other SRLs, included gastrointestinal (GI) discomfort, diarrhea, nausea, and vomiting. Thirty-five percent of study participants developed gallbladder or biliary related adverse effects, most commonly cholelithiasis, but a minority underwent cholecystectomy. Transaminases elevation was usually less than three times ULN (28, 30, 32).

Rare instances of QTc prolongation were also observed and clinicians should be aware to avoid potential drug interactions further increasing QT. This may also limit potential therapeutic drug-drug combinations in treating CD (e.g., combination with ketoconazole or mifepristone may lead to additive QT prolongation effects). However, in an animal model study, QTc prolongation in combination with osilodrostat was not observed (43).

## Monitoring Therapy

Monitoring of CD patients’ therapy is challenging. Petersenn et al. demonstrated that intra-individual UFC samples may have up to 50% variability, and that variability increased as absolute UFC values also increased (44). Moreover, no correlations exist between UFC and clinical features of hypercortisolism (44). LNSC follow-up seems to be the most accurate compared with UFC, serum cortisol and plasma ACTH in predicting postoperative recurrence in CD (19, 45, 46). In a sub-analysis of pasireotide (short-acting) phase III trial, LNSC and UFC concentrations were positively correlated to each other at baseline (*r* = 0.97). Pasireotide decreased LNSC levels during 12 months of treatment and it was a moderate correlation (*r* = 0.55) between individual patient LNSC and UFC values when all time points on treatment were pooled (47). Multiple LNSC values can possibly be used and are more convenient to analyze long-term response to pasireotide in lieu of multiple UFC tests, provided LNSC was high at baseline (4).

## Medications Approved for Other Indications than CD

### Cabergoline

Dopamine is a catecholamine important for neurotransmission, for vasoregulatory effects, and for other neurological functions (21, 48). Dopamine receptors are G-protein-coupled receptors consisting of five subtypes, which are further classified into D1-like stimulating receptors (D1, D5) and D2-like (D2, D3, D4) inhibiting receptors. D2 is expressed in approximately 80% of corticotroph adenomas and its expression seems a *sine qua non* condition for response to dopamine agonist (49).

Cabergoline is the dopamine agonist (DA) of choice given its long plasma half-life, its high affinity for the D2 receptor, and is better tolerated compared with bromocriptine (50). Usual doses in CD are 1.5–5 mg/week and based on initial studies, response to cabergoline is expected to be 30–50% in patients with mild or moderate disease in the short term (51–54). This number drops by half in prolonged studies as 20–25% persist with response at 2–3 years (52, 55). Moreover, a recent prospective study on cabergoline used as a first-line therapy reported a disappointing partial response rate (defined as more than 50% decrease in UFC) of 25% at 6 weeks; only 10% had an improvement in both LNSC and UFC (56). These findings were confirmed by a large retrospective study that included 53 patients treated with cabergoline monotherapy. Forty percent of patients had normalized UFC with clinical improvement in the first 12 months on therapy. On long-term follow-up, 28% of responders stopped treatment because of loss or response or intolerance (55).

Common side effects include orthostatic hypotension due to dopamine’s vasodilatory effects, nausea, headache, and dizziness. Dopamine agonist associated cardiac valvulopathy has been reported, when cabergoline was mostly used to treat Parkinson’s disease (PD) (57). Doses used in CD are lower than in PD but higher than prolactinomas (55, 58); other series in prolactinomas have not found a relation (51, 52, 58, 59), but precaution is advised.

Cabergoline is advantageous for women in or planning pregnancy where therapeutic options are otherwise limited. Its innocuous profile during gestation has been confirmed with an increasing number of uneventful pregnancies (mostly for prolactinomas) (60). Efficacy and safety of cabergoline in CD in pregnancy is also supported by a number of case reports (61–63).

### Combination Therapy

Most corticotroph adenomas co-express SSTR5 and D2 receptors, and combining pasireotide and cabergoline could have additive or even synergistic effects (21, 64). An open-label multicenter study followed 66 patients initiated on pasireotide and receiving add-on cabergoline if not controlled on 900 μg twice-daily monotherapy. Two-thirds (39/66) of patients required combination therapy and cabergoline allowed a third of those (13/39) to normalize mean UFC after 35 weeks (65) with no additional safety signals.

Pasireotide and/or cabergoline regimens in combination with adrenal-steroidogenesis inhibitors have also been used (23, 55, 66). In a small study by Vilar et al., adding ketoconazole in patients uncontrolled on cabergoline monotherapy has been shown to normalize UFC values in two-thirds of patients (66). Feelders et al. examined combination therapy of pasireotide with cabergoline and ketoconazole in an open-label prospective study with 17 participants (67). Although the study did demonstrate improvement in UFC normalization with the addition of cabergoline to pasireotide (from 29 to 53%), the patients included in the study had significantly lower mean baseline UFC levels compared with the phase III subcutaneous pasireotide trial, thus possibly underrepresenting the potential added therapeutic effects of cabergoline to pasireotide.

This approach may allow smaller doses thus minimizing side effects without compromising efficacy and is an option for patients not achieving control or intolerant (19, 68).

Although no QTc prolongation has been seen in an animal model study (43), there are no data on patients treated with combination using novel steroidogenesis inhibitors such as osilodrostat and previously described pituitary-directed therapies.

### Temozolomide

This oral alkylating agent is also used in the treatment of other cerebral neoplasms because of its ability to cross the blood-brain barrier easily (69). Temozolomide can be used in selected cases of aggressive CD caused by macroadenomas, where management of a locally progressive disease is also a concern (69–71). Temozolomide induces cell apoptosis by methylating DNA, specifically at guanine at 06-position, thus interfering with the next DNA replication cycle (69). At the cellular level, this chemotherapeutic agent induces better cell differentiation and a reduction in proliferation, measured by decrease mitosis and Ki-67 index (71). Repair enzyme 06-methyl-guanine DNA methyltransferase (MGMT) reverses alkylation of the guanine and potentially cancels drugs effect. MGMT, evaluated by pathologists, has been suggested as a predictive factor and low MGMT has been associated with a positive response, but is not universally observed (69–71).

The most commonly used regimen is 150–200 mg/m^2^/day for 5 days each month (total cycle dose of 750–1,000 mg/m^2^). Patients must be monitored for cytopenias (mainly neutropenia and thrombopenia), and dose reduction or interruption may be warranted. Common side effects include GI issues, headache, hearing loss and dizziness. In cases of invading tumors, cerebrospinal fluid leak may occur with tumor shrinkage (72). In gliomas, temozolomide has synergistic effects with radiotherapy, both altering DNA and interfering with cell replication process (73). This has not directly been studied in pituitary tumors but could potentially be effective.

Temozolomide is used either alone, or in combination therapy with pasireotide (74). Partial or complete response is usually around 80% (71). Clinical response to temozolomide is rapid, and in cases of CD, structural and biochemical improvement can be observed within 2 months of therapy (69–71). At macroscopic level, tumors become soft and friable and can present as hemorrhage, necrosis and cystic changes on imaging (69). Adjuvant temozolomide pre-surgery has not been studied, although it can be theoretically of benefit in large aggressive tumors if preoperative shrinkage is achieved.

Medical treatment options (available and on the horizon) are summarized in Table 2 (28, 30, 31, 55, 71, 75–80), and their respective mechanism of action is depicted in Figure 1.

**Table 2:** Summary of pharmacologic therapies for Cushing’s disease (28, 30, 31, 55, 71, 75–80).

Name	Dose	Route	Mechanism of action	Efficacy to normalize urine free cortisol (%)	Responders characteristics	Tumor size reduction	Side effects	Comments
Pasireotide	600–900 μg twice daily	Subcutaneous	Agonist SSTR5 > 2	30–40	UFC < 5× ULN	25–80%	GI and biliary issues Hyperglycemia QT prolongation	Only drug approved for CD
Pasireotide LAR	10–30 mg monthly	Intramuscular		30–50	UFC < 2× ULN	10–20%		

Cabergoline	0.5–6 mg weekly (in divided doses)	Oral	Dopamine agonist (D2)	25–40	Small subgroup of corticotrophs adenomas expressing D2 receptor	N/A	Hypotension Nausea Headache	Usually short-term response

Temozolomide	150–200 mg/m^2^/day ×5 days monthly	Oral	Methylation DNA	80	Possibly patients with negative MGMT mutation	0 (stable)–50% for most patients; rarely patients had progressive tumor growth	GI issues Headache Dizziness Hearing loss	Aggressive adenomas or carcinomas

Roscovitine	400 mg twice daily	Oral	Inhibition CDK/cyclin E	N/A	N/A	N/A	Preliminary: Asthenia Nausea Vomiting Hypokaliemia	Phase II study ongoing

Retinoic acid	80 mg once a day	Oral	Agonist RAR	25	Absence of COUP-TF1	N/A	Mucositis Photosensitivity Hypertriglyceridemia	Based on small studies

Gefitinib	250 mg once a day	Oral	Inhibition EGFR	N/A	USP8-mutated adenomas	N/A	Skin reaction Diarrhea Interstitial pneumonitis	Phase II study ongoing

Silibinin	To be determined	To be determined	Inhibition HSP90	N/A	N/A	N/A	Minimal	Animal studies only

*N/A, not available; HSP90, heat shock protein 90; USP8, ubiquitin-specific protease 8; EGFR, epidermal growth factor receptor; CDK, cyclin-dependant kinase; UFC, urinary free cortisol; ULN, upper limit of normal; RAR, retinoic acid receptor; COUP-TF1, chicken ovalbumin upstream promoter transcription factor-1*.

## Drugs in Development for CD

### Roscovitine

Roscovitine is an oral cyclin-dependant kinase (CDK) 2/cyclin E inhibitor (also called R-roscovitine, seliciclib, or CYC202). CDKs are involved in cycle cell progression, differentiation and transcription. During the corticotroph cell cycle, the activated complex formed by CDK2 and cyclin E normally activates pro-opiomelanocortin (POMC) transcription and cell proliferation with decrease cell senescence (78). Corticotroph adenomas uniquely overexpress cyclin E causing ACTH hypersecretion (78), therefore potentially making CDK2/cyclin E a very precise therapeutic target.

Roscovitine is a purine analog acting by direct competition for ATP-binding sites causing inhibition of CDK2 but also CDK 1, 5, and 7 (81, 82). It has been observed *in vitro* in zebrafish embryos and murine model to inhibit ACTH secretion and to induce cycle cell arrest (83). *In vitro* human pituitary corticotroph tumors treated with roscovitine showed marked reduction in ACTH levels, and a lesser inhibitory effect on tumor growth (78). In USA, this agent is under phase II study at 400 mg bid for 4 weeks (84).^2^

Roscovitine is also being studied in a multitude of cancer disease including breast and lung (82, 85) and in other diseases such as pain syndromes and nephritis. Data on safety are yet to be documented, but phase I clinical trials reported that asthenia, nausea, vomiting and hypokalemia are the most frequent side effects, mostly observed at doses above 800 mg twice daily (82).

### Retinoic Acid (RA)

Vitamin A derivatives, mainly RA, are important modulators of cell proliferation and function on target organs such as skin, eyes, and brain. RA binds to its nuclear receptor and forms a heterodimer with RA and retinoid X receptors (each having alpha, beta, and gamma subtypes) engendering transcriptional effects on target genes (86). Retinoic acid receptor alpha exists in hypothalamic paraventricular nucleus with corticotropin-releasing hormone and arginine vasopressin, but its precise physiological role is uncertain (86). In the hypothalamus–pituitary–adrenal axis, glucocorticoid receptor (GR) activation depends on its phosphorylation status and is modulated by RA. When tested on murine models of pituitary corticotroph tumors, RA has an inhibitory effect (87, 88). RA causes both inhibition of ACTH secretion *via* POMC gene transcription mediated by AP-1 and Nur77 (87), and inhibition of corticotroph growth with increase cell death mediated by caspase-3-activity (87). Interestingly, RA action seems specific to adenomatous cells not affecting normal pituitary. More specifically, normal pituitary cells express chicken ovoalbumin upstream promoter transcription factor 1 (COUP-TF1), an orphan nuclear receptor, which prevents inhibition from RA (87). Expression of COUP-TF1 in tumoral cells seems rare, and its presence could be a reason for RA non-response. In a characterization study, only 15% of corticotroph tumors expressed COUP-TF1; all were macroadenomas (75). In addition to its central action, a dual peripheral effect is observed and antiproliferative effect is observed at the adrenal level, mediated by bone morphogenic protein 4 action.

Animal studies showed biochemical and clinical benefit in dogs with CD treated with RA (89) and RA inhibited ACTH production and proliferation indexes in corticotroph cell cultures (87). RA also decreased by approximately 60% the expression of melanocortin receptor type 2 (MC2R), the adrenal ACTH receptor in adrenal cell cultures (90).

Pecori Giraldi et al. showed five out of seven CD patients treated at doses of 80 mg daily for 6–12 months had favorable responses, in which UFCs decreased from 20 to 70% and normalized in three patients (77). Clinical improvement in features of hypercortisolism was also observed (77). Interestingly, ACTH levels initially decreased then returned to initial values without a loss of efficacy. Another small study on CD patients showed that 4/16 (25%) achieved normal UFC levels after 12 months of treatment with 13-*cis*-RA (e.g., isotretinoin, an isomer of RA) (91).

Retinoic acid is used in different diseases such as severe acne vulgaris and hematologic diseases, and undesirable effects can be minimized by prevention and close monitoring. Frequent side effects are usually manageable such as photosensitivity, cheilitis, mucositis, and hypertriglyceridemia (91, 92). Most serious teratogenic effects of RA are well known and in young women with CD, a contraceptive method is essential. Doses studied in CD are similar than those used for acne (92), and therefore the side effect profile should be similar. If combination therapy is entertained, clinicians also must consider potential drug interactions with ketoconazole that will inhibit isotretinoin metabolism.

### Epidermal Growth Factor Receptor (EGFR) Inhibitors

Epidermal growth factor receptor is a cell surface receptor member of the ErbB family. Specific ligands such as EGF or other growth factors can stimulate tyrosine kinase activity with downstream activation of MAPK pathway, phosphatidylinositol-3-kinase, phospholipase C gamma, and transcription factors leading to cell proliferation and differentiation. EGFR overexpression leads to upregulated cell proliferation and is linked with many cancers (93). In CD, EGFR activation also induces high levels of POMC expression and transcription (76). Notably, EGFR is highly expressed in corticotroph tumors (up to 75%) and correlates with a more aggressive subtype (8). EGFR overexpression is linked to downregulated p27, a CDK inhibitor implicated in cell cycle which is depleted in CD and restored by EGFR blockade (76, 94).

The genetic basis of elevated EGFR signaling in CD has recently been clarified by whole genome sequencing. EGFR overexpression is linked to somatic mutation in the candidate ubiquitin-specific protease 8 (USP8) gene (95, 96). USP8 mutation is found in 30–60% of ACTH tumors (95, 96) and appears specific to corticotroph tumors (96). Mutated USP8 adenomas harbor more EGFR expression, higher EGFR protein and higher POMC mRNAs (96). USP8 mutation at the 14-3-3 binding motif leads to a hyperfunction of this deubiquinating enzyme directed to its target EGFR leading to reduce EGFR degradation (95, 96). USP8 knockdown leads to reduce EGFR protein and inhibits ACTH secretion. USP8 characterization is not widely available, but USP8-mutated corticotroph adenomas causing CD appear more frequently in women (70–95% females), are smaller in size (most tumors are <0.5 cm) and are less radiologically invasive, compared with wild type (95, 97). Of note, 30–40% of these tumors were also very large and invasive (97). Biochemical parameters such as ACTH levels of degree of hypercortisolism were similar, but the secreting ability of USP8-mutated tumors was proportionally higher (e.g., higher ACTH levels compared with size) than other corticotroph adenomas (95, 97). These clinical characteristics may help identify tumors harboring USP8 mutations pending widely available genetic testing.

Blocking EGFR induces reduced POMC promoter activity with antiproliferative effects in a dose-dependent manner in cultures of canine and human corticotroph tumors (76). Gefitinib, an oral EGFR inhibitor, is actively studied in USP8-mutated CD in China (phase II) (98).^3^ *In vivo* gefitinib treatment of mice with corticotropinomas (AtT20 allografts) both led to decreased tumor volume and clinical improvement in omental fat (76). Gefitinib is indicated for the first-line treatment of patients with metastatic non-small-cell lung cancer with tumors harboring EGFR-specific mutations (93). Used at doses of 250 mg daily, gefitinib’s most frequent side effects were Grade 1 or 2 skin rash and diarrhea, mild elevation of transaminases and rare development of interstitial lung disease (2.6% Grade > 3), for which monitoring is advised (99).

## Future Possible Targets

### Genetic Manipulation of MicroRNA (miRNA)

MicroRNAs are small non-coding gene portions that have an important role in regulating other genes expression. Evidence exists in the cancer field that inhibition of miRNAs could be achieved by an antisense oligomer delivered to the tumor (100). This evolving area of therapy may be applicable to pituitary tumors. miRNAs are implicated in pituitary tumor growth, differentiation and aggressiveness by modulating transcription factors or enzyme activity by methylation or by other mechanism (101). In corticotroph adenomas, there is downregulation of several miRNAs, losing their tumor suppressive action. Stilling et al. found that miRNA-122 and -493 are implicated in tumor development of aggressive corticotroph adenomas and carcinomas (102). Their manipulation could potentially lead to tumor regression.

### Silibinin; Heat Shock Protein 90 (HSP90) Inhibition

Heat shock protein 90 is a chaperone protein interacting with the GR to induce conformation changes essential for GR action, thereby regulating cell proliferation and POMC transcription. HSP90 allows proper folding of the GR ligand-binding domain. Once cortisol binds to its surface receptor, the unit then translocates to the nucleus where NR3C1 binds to regulatory areas on DNA, affecting transcription of genes implicated in synthesis of POMC, consequently inhibiting ACTH secretion (8). This cascade is the basis of the glucocorticoid (GC) negative feedback inhibition. HSP90 is overexpressed in corticotroph adenomas, leading to its continued binding to GR, consequent decreased sensitivity of GR to circulating GC (and dexamethasone, for example), and ultimately increased transcription of POMC (79).

Heat shock protein 90 inhibitors cause release of the GR from HSP90. This might restore GC sensitivity by stabilizing GR in a high-affinity conformation for ligand binding (103). N- and C-terminal inhibitors have been studied and act at different levels of the cycle. The most promising agent is silibinin, a C-terminal HSP90 inhibitor extracted from milk thistle seeds. In a murine corticotroph cell line (AtT20), silibinin increased the transcriptional activity of NR3C1 and GR sites, leading to decrease POMC transcription and suppression of ACTH secretion (79). This effect was enhanced by dexamethasone. Mice with corticotroph tumors treated with silibinin showed decreased tumor growth, lower hypercortisolism levels and clinical improvement in their cushingoid features (79).

Silibinin has a very favorable side effect profile, and milk thistle herbal supplement is mostly used for liver and biliary issues (104, 105). Silibinin has been studied for its anti-fibrotic hepatic effect (106), but also for its anti-cancerous and anti-inflammatory action in prostate cancer, Alzheimer disease and osteoarthritis (107–109).

### Testicular Orphan Receptor 4 (TR4) Inhibition

Testicular orphan receptor 4 is a nuclear receptor and transcription factor implicated in many physiological processes (103). TR4 is normally expressed in corticotroph cells, and TR4 knockdown leads to decreased POMC expression (110). Overexpression of TR4 has been observed in corticotroph tumors, thereby increasing POMC transcription and ACTH secretion, but also cell proliferation (110). These actions are also based on decreased sensitivity to circulating GC, as TR4 blunts the interaction between GC and GR with POMC gene promoter (111). Increased activity of TR4 could result from EGFR signaling, thus also potentially linking its expression to USP8 (111). These findings make TR4 a potential therapeutic target, but no antagonist or inhibitor has been developed to date.

### Monoclonal ACTH Antibody

ALD1613 is a specific, high-affinity, monoclonal antibody-blocking ACTH signaling. This molecule binds to ACTH with high-affinity and high-specificity thus inhibiting MC2R signaling *in vitro*. It neutralized ACTH activity *in vitro* and reduced GC levels in rats and in cynomolgus monkeys (112). ALD1613 treated rats had also a blunted GC response to stress (112). There are no published human studies to date.

## Conclusion

With increased knowledge of CD pathophysiology (113) at the molecular level, the face of medical therapy for CD is quickly changing. Pasireotide remains the only drug approved specifically to treat CD at the pituitary level, but the pharmacopeia continues to expand. Future studies as to precise efficacy and safety of new pharmaceutical agents and identification of response predictors, are in the wings. A multidisciplinary team with collaborating experts remains the basis of complex disease management. A personalized and individual-based approach is key to offering treatment with highest success rate while minimizing the side effects and improving patient quality of life.

## Author Contributions

FL contributed to conception and design, acquisition of data, analysis and interpretation of data, drafting, critically revising, statistical analysis, and administrative/technical/material support, and reviewed submitted version of manuscript. JC contributed to acquisition of data, analysis and interpretation of data, drafting, critically revising, statistical analysis, and administrative/technical/material support, and reviewed submitted version of manuscript. MF contributed to conception and design, acquisition of data, analysis and interpretation of data, drafting, critically revising, statistical analysis, administrative/technical/material support, and study supervision, and reviewed submitted version of manuscript.

## Conflict of Interest Statement

MF has received scientific consultant fees from Novartis Pharmaceuticals and Strongbridge Biopharma and is a principal investigator in clinical trials sponsored by Novartis Pharmaceuticals, Millendo Therapeutics, and Strongbridge Biopharma, with research support to OHSU. FL and JC have no competing interests.

## Footnotes

^1^https://clinicaltrials.gov/ct2/show/NCT02060383 (Accessed: January 2, 2018).

^2^https://clinicaltrials.gov/ct2/show/NCT02160730 (Accessed: January 2, 2018).

^3^https://clinicaltrials.gov/ct2/show/NCT02484755 (Accessed: January 2, 2018).
